# Acute responses to low-intensity aerobic exercise with continuous and intermittent blood flow restriction

**DOI:** 10.3389/fspor.2025.1737095

**Published:** 2026-01-15

**Authors:** James Brown, Jakob D. Lauver, Timothy R. Rotarius, Justin P. Guilkey

**Affiliations:** 1 Department of Kinesiology, Coastal Carolina University, Conway, SC, United States; 2 School of Kinesiology, Ball State University, Muncie, IN, United States

**Keywords:** cycling, interval exercise, myocardial work, near-infrared spectroscopy, tissue oxygen saturation, venous occlusion

## Abstract

**Introduction:**

This study examined muscular and cardiovascular responses to light-intensity aerobic exercise with different blood flow restriction (BFR) protocols.

**Methods:**

Ten males performed four protocols on the cycle ergometer: low-intensity exercise with no BFR (LIE), LIE with continuous BFR (CONT-BFR), LIE with intermittent BFR (INT-BFR), and high-intensity exercise (HIE). Each protocol consisted of five 2-min work intervals (INT) at 35% of peak work rate for LIE, CONT-BFR, and INT-BFR and 70% of peak work rate for HIE. During CONT-BFR, cuffs were inflated to 60% of arterial occlusion pressure (AOP) at the start of INT 1 and remained inflated until the end of INT 5. During INT-BFR, cuffs were inflated to 60% of AOP during INTs and deflated during recovery intervals. Tissue oxygen saturation (StO_2_) and rate pressure product (RPP) were measured to assess hypoxic stimulus and myocardial work, respectively.

**Results:**

StO_2_ response was similar between CONT-BFR (−23.2 ± 12.4 change from baseline arbitrary units ΔBSL AU), INT-BFR (−23.1 ± 13.1 ΔBSL AU), and HIE (−26.1 ± 13.1 ΔBSL AU); StO_2_ response was the smallest in LIE (−2.8 ± 13.0 ΔBSL AU). RPP was not different between CONT-BFR (20,872.8 ± 2,393.3 mmHg·bpm) and INT-BFR (21,056.7 ± 2,701.5 mmHg·bpm), but both were lower than HIE (30,760.2 ± 1,729.1 mmHg·bpm). RPP during LIE (17,893.2 ± 2,202.6 mmHg·bpm) was lower than all protocols.

**Discussion:**

There were no differences in hypoxic stress or myocardial work between BFR conditions. BFR conditions, regardless of restriction protocol, produced similar hypoxic stimulus with lower cardiac work compared to HIE; BFR protocol could be an alternative to HIE.

## 1 Introduction

Exercise with blood flow restriction (BFR) utilizes a tourniquet or pressurized cuff to externally compress the limb proximal to the exercising muscle reducing arterial blood inflow and venous outflow during low-intensity exercise (1). Low-intensity aerobic training with BFR has been shown to increase muscle strength and hypertrophy, in addition to improving aerobic fitness and endurance performance (2–4). The mechanism for positive adaptations during low-intensity exercise with BFR is not entirely known, but a prominent contributor is thought to be increased muscle hypoxic stimulus compared to free-flow conditions (5–7). Takarada et al. (7) showed muscular hypertrophy was greater following low-load resistance training with BFR compared to low-load resistance training under free-flow conditions and similar to that of high-load resistance training without BFR. In addition to muscular adaptations, cardiovascular responses should be considered in BFR protocols to safely apply the practice (1). While adding blood flow restriction (BFR) to low-intensity exercise is generally considered safe for most populations (1, 8), it does increase myocardial workload. This is reflected in a higher rate-pressure product (RPP), driven by increases in heart rate and systolic blood pressure compared to free-flow conditions at similar intensities (5, 9, 10). Therefore, BFR protocols should try to balance the hypoxic stimulus and cardiovascular work to maximize adaptation and minimize cardiovascular stress.

Previous research has indicated that differences in the application of BFR may affect acute exercise responses (11–13). Indeed, changes in both exercise intensity with BFR (11) and restriction pressure (12, 13, 39, 40) can lead to varying levels of muscle hypoxia and cardiac work during resistance and aerobic exercise. In addition to this, the timing and duration of restriction may also alter the acute responses (14, 15). For example, recent research (16) has extended BFR application into the post-exercise phase of sprint-interval training, demonstrating greater improvements in muscle oxygen extraction and power output at the gas exchange threshold after four weeks compared to free-flow sprint-interval training. These enhanced endurance-related adaptations appear to result from the amplified hypoxic stimulus during the post-exercise BFR period, underscoring how timing strategies can influence physiological outcomes. However, despite these advances, research directly comparing intermittent (INT-BFR) and continuous (CONT-BFR) protocols during high-intensity interval aerobic exercise remains limited (1). In CONT-BFR, cuffs remain inflated during both exercise and rest intervals, resulting in prolonged restriction. This extended duration of restriction may heighten hypoxic stress, but also, amplify the pressor reflex and cardiovascular demand, due to limited metabolite clearance (8). In contrast, INT-BFR pressure is applied only during work intervals, allowing reperfusion during recovery intervals. This approach may reduce the hypoxic stimulus and cardiovascular work (13, 15, 17, 18).

Studies by Corvino et al. (13, 41) appear to be the only two studies that have examined physiological responses to INT-BFR and CONT-BFR during aerobic exercise. Both studies found a greater decrease in muscle oxygenation during INT-BFR compared to CONT-BFR. However, restriction pressure was greater during INT-BFR (20 mmHg greater than the arterial occlusion pressure) compared to CONT-BFR (80% of arterial occlusion pressure), in both studies. Therefore, it is unclear if the greater hypoxic stress was due to the restriction protocol or the restriction pressure. Moreover, these studies only examined the effect of the restriction protocol on hypoxic stress. Cardiovascular responses to BFR during aerobic exercise remain underexplored. Evidence suggests that BFR increases hemodynamic load compared to exercise alone (2) and can elevate heart rate, cardiac output, and myocardial workload. These responses may have implications for safety and prescription, particularly in healthy populations. However, it appears no studies have examined the effect of restriction protocol during aerobic exercise on cardiovascular work. As such, the effect of restriction protocol (INT-BFR and CONT-BFR) during low-intensity aerobic exercise is unclear.

Understanding acute hypoxic stress produced by INT-BFR and CONT-BFR at the same restriction pressure could give guidance in developing effective BFR training protocols to maximize adaptation. Moreover, early reviews [e.g., (19, 20)] have highlighted the incomplete understanding of cardiovascular responses to BFR, this study aims to address this gap by comparing INT-BFR and CONT-BFR during aerobic exercise. Cardiovascular responses to INT-BFR and CONT-BFR in healthy adults could give insight into the safety of each protocol and the prescription of protocols for healthy populations. This knowledge can help practitioners select the safest and most effective protocol for potential populations that cannot perform high-intensity exercise. Therefore, the primary purpose of this study was to examine the muscle hypoxic response and cardiovascular responses to low-intensity interval exercise with INT-BFR and CONT-BFR, low-intensity interval exercise without BFR (LIE), and high-intensity interval exercise without BFR (HIE). It was hypothesized that the muscle hypoxic stress and cardiac work would be greater during CONT-BFR compared to INT-BFR due to the continuous restriction of blood flow to the muscle and occlusion of venous outflow compared to intermittent free-flow conditions. Additionally, the hypoxic stress and cardiac work in both BFR conditions would be greater than LIE but less than HIE.

## 2 Materials and methods

### 2.1 Participants

Male participants between the ages of 18 and 45 years, who were not engaged in high-intensity interval exercise or BFR training prior to participation, were recruited for this study. Based on their responses to a health history questionnaire, all participants had no diagnosed with cardiac, pulmonary, or metabolic disease, and were not taking medications that influenced cardiovascular responses. Prior to participation in the study, all participants were advised of the risks and benefits of participation in the study and gave written consent. The study complied with the standards set by the Declaration of Helsinki and was approved by the university's Institutional Review Board.

Power calculations were performed using the G*Power software (21) using effect sizes from previous studies that examined hemodynamic and muscle oxygenation during intermittent and continuous BFR (13, 14). For StO_2_, the absolute standardized mean differences across significant protocol contrasts reported by Corvino et al. (13) derived an effect size of d = 0.95. Effect sizes in Neto et al. (9) were larger (HR d = 1.608), and the effect size for RPP could not be determined. Using the most conservative effect size indicated that a sample size of 9 participants would be sufficient when α = 0.05 and β = 0.90 for the repeated-measures design. This sample size is similar to studies that have examined muscle oxygenation and blood pressure during exercise with BFR (5, 11, 12, 14).

### 2.2 Experimental protocol

This study consisted of five visits to the laboratory performed at approximately the same time of day; each visit was separated by at least 48 h, but no more than seven days. During the first visit, maximal exercise responses were determined from a 20 watt per minute ramp test to volitional exhaustion on a cycle ergometer (Corival, Lode, The Netherlands). In lieu of a plateau in VO_2_, secondary criteria were used to establish a maximal effort. The criteria used were as follows: (1) failure to maintain a pedal rate greater than 50 rpm, (2) RER ≥ 1.1, and (3) peak HR ≥ 85% of age-predicted maximal (208–0.7 × age) (22). If participants achieved all three criteria a maximal effort would be achieved; if not they would be asked to repeat the test on a separate day. Breath-by-breath gas exchange was collected continuously and averaged over 15 s by a metabolic cart (Quark PFT, Cosmed, Rome, Italy). Peak oxygen consumption (VO_2peak_) and peak power (P_peak_) were the highest 15-second VO_2_ and highest power achieved during the test. P_peak_ was used to determine the work rates for experimental conditions.

The second, third, fourth, and fifth visits served as experimental conditions. The experimental conditions used a repeated-measures crossover design, in which each subject performed four experimental conditions: HIE, LIE, INT-BFR, and CONT-BFR. Experimental conditions were assigned in a randomized order (http://www.randomization.com).

### 2.3 Exercise protocols

Each protocol commenced with a 4-minute warm-up at 20 W, followed by five 2-minute work intervals (INT) with 1-minute recovery intervals (REC at 20 W) between each work interval. The 2:1 work-to-recovery ratio was selected based on previous studies in our laboratory (11) and others (1, 13). The INTs during LIE, INT-BFR, and CONT-BFR were 35% P_peak_ and INTs were 70% P_peak_ during HIE. For CONT-BFR, cuffs were rapidly inflated at the start of the first INT and remained inflated until the end of the fifth INT. For INT-BFR, the cuffs were rapidly inflated at the start of the first INT and deflated at the start of the first REC. This was repeated for all INTs until the cuffs were deflated at the end of the fifth INT. For both BFR exercise protocols, the cuffs were inflated to 60% of the arterial occlusion pressure (AOP). The AOP percentage was based on previous research, which has shown similar hypoxic stress during resistance exercise with 60% or 80% AOP (39). Pilot work found that 80% of LOP was too high a restriction pressure for all participants to complete the CONT-BFR trial.

### 2.4 Arterial occlusion pressure

To determine the restriction pressure during INT-BFR and CONT-BFR, AOP was determined prior to each exercise protocol with BFR by the same investigator. Cuffs (Hokanson, SC10D, Bellevue, WA, 10.0 cm width) were placed bilaterally on the proximal portion of the thighs. AOP was determined in a supine position following a five-minute supine rest. The posterior tibial artery on the right leg was located using Doppler auscultation (Nicolet, Imex Pocket Dop II) and the cuffs were inflated. The pressure was slowly increased using a rapid inflation device (Hokinson, Bellevue, WA) until the pulse ceased. The pressure at which the pulse was no longer audible was considered the AOP.

### 2.5 Measurements

During all experimental conditions, breath-by-breath gas exchange was recorded using a metabolic measurement system (Quark PFT, Cosmed, Rome, Italy). Additionally, heart rate (HR), stroke volume (SV), and cardiac output (CO) were collected continuously via impedance cardiography (Enduro, Physioflow, France, (HR ICC = 0.84–0.98; SV ICC = 0.78–0.97; CO ICC = 0.70–0.96) (23). VO_2_, HR, SV, and CO were averaged over the final 30 s of the warm-up (WU), INT 1, INT 3, and INT 5.

Tissue oxygen saturation (StO_2_) was collected using a single-channel continuous-wave near-infrared spectroscopy (NIRS) monitor (Moxy Muscle Oxygen Monitor, Fortiori Design LLC, Minnesota, USA, StO₂ ICC = 0.773–0.92) at a frequency of 0.5 Hz (24, 25). The NIRS device was covered with a shield to limit external light sources and affixed midway between the anterior iliac spine and the superior border of the patella over the belly of the vastus lateralis (VL) on the right leg. During the first visit, VL skinfold thickness was measured in duplicate at the site where the NIRS device was placed to estimate subcutaneous fat, as adipose tissue can affect light penetration and influence NIRS measurements. StO_2_ was averaged over the final 30 s of the WU and INTs and the final 15 s of the REC. Because continuous-wave NIRS monitors cannot determine absolute concentrations, reported StO_2_ data are expressed as a change from the baseline data (ΔBSL) in arbitrary units (AU). The baseline data was the average StO_2_ over the final 30 s of the WU. Additionally, the overall hypoxic stress of each protocol was determined by calculating the StO_2_ area under the curve (AUC). Normalized StO_2_ values from the start of INT 1 through the end of INT 5 were plotted against time and StO_2_ AUC was calculated via the trapezoidal rule.

Systolic (SBP) and diastolic (DBP) blood pressure was also taken by manual auscultation during the final 30 s of the WU, INT 1, INT 3, and INT 5. The same investigator measured blood pressure through all experimental conditions in the same participant. RPP was calculated by multiplying the HR (bpm) by the SBP (mmHg).

### 2.6 Statistical analysis

Statistical analyses were completed on IBM SPSS statistical software (Version 25.0; SPSS, Inc., Chicago, IL). A Shapiro–Wilk test was performed to determine the normality of all variables. A two-way (condition by time) repeated-measures ANOVA was used to compare the StO_2_ change scores (ΔBSL) overall INTs and RECs between conditions. A one-way repeated measures ANOVA was used to compare StO_2_ AUC between conditions A two-way (condition by time) repeated-measures ANOVA was used to compare the VO_2_, HR, SV, CO, SBP, DBP, and RPP responses during WU, INT 1, INT 3, and INT 5 between conditions. Test–retest reliability during WU across four visits was assessed using intraclass correlation coefficients (ICC) calculated with a two-way mixed-effects model and an absolute agreement definition. Average-measure ICC values were reported for each assessment, with interpretation based on standard thresholds (poor <0.50, moderate 0.50–0.75, good 0.75–0.90, excellent >0.90).

In response to significant interactions, subsequent pairwise *post-hoc* comparisons with a Bonferroni correction were made to compare responses between conditions at each time point. Partial eta squared (*η*_p_^2^) was used to evaluate the effect of significant ANOVA analyses. Cohen's d estimated of effect size of significant pairwise comparisons (small effect < 0.4, medium effect = 0.40–0.75, large effect = 0.75–1.1, very large effect = 1.1–1.45, and huge effect > 1.45) (26). Statistical significance was established if *p* ≤ 0.05.

## 3 Results

### 3.1 Subject characteristics

Ten male participants (age = 28.0 ± 9.0 years old, height = 171.9 ± 20.4 cm, weight = 76.6 ± 10.8 kg, VL skinfold thickness = 22.4 ± 6.8 mm) completed all study conditions. Although no participants achieved a plateau in VO₂ during the test, all met the three secondary criteria for maximal effort. The participant's VO_2peak_, HR_peak,_ and RER_peak_ were 2.52 ± 0.37 L·min^−1^ (33.3 ± 5.3 mL·kg^−1^·min^−1^), 183 ± 11 bpm, and 1.42 ± 0.08, respectively. The *P_peak_* was 238.8 ± 33.4 W. This calculated work rate for INT was 166.7 ± 22.8 W during HIE and 83.2 ± 11.3 W during LIE, CONT-BFR, and INT-BFR.

Before CONT-BFR, AOP was 201.4 ± 28.3 mmHg; cuffs were inflated to a restriction pressure of 120.9 ± 16.8 mmHg during the exercise protocol. Prior to INT-BFR, AOP was 201.9 ± 29.4 mmHg and cuffs were inflated to a restriction pressure of 121.2 ± 17.6 mmHg during INTs. Differences in AOP and restriction pressure between conditions were not significant.

### 3.2 Tissue oxygenation

**Figure 1:**
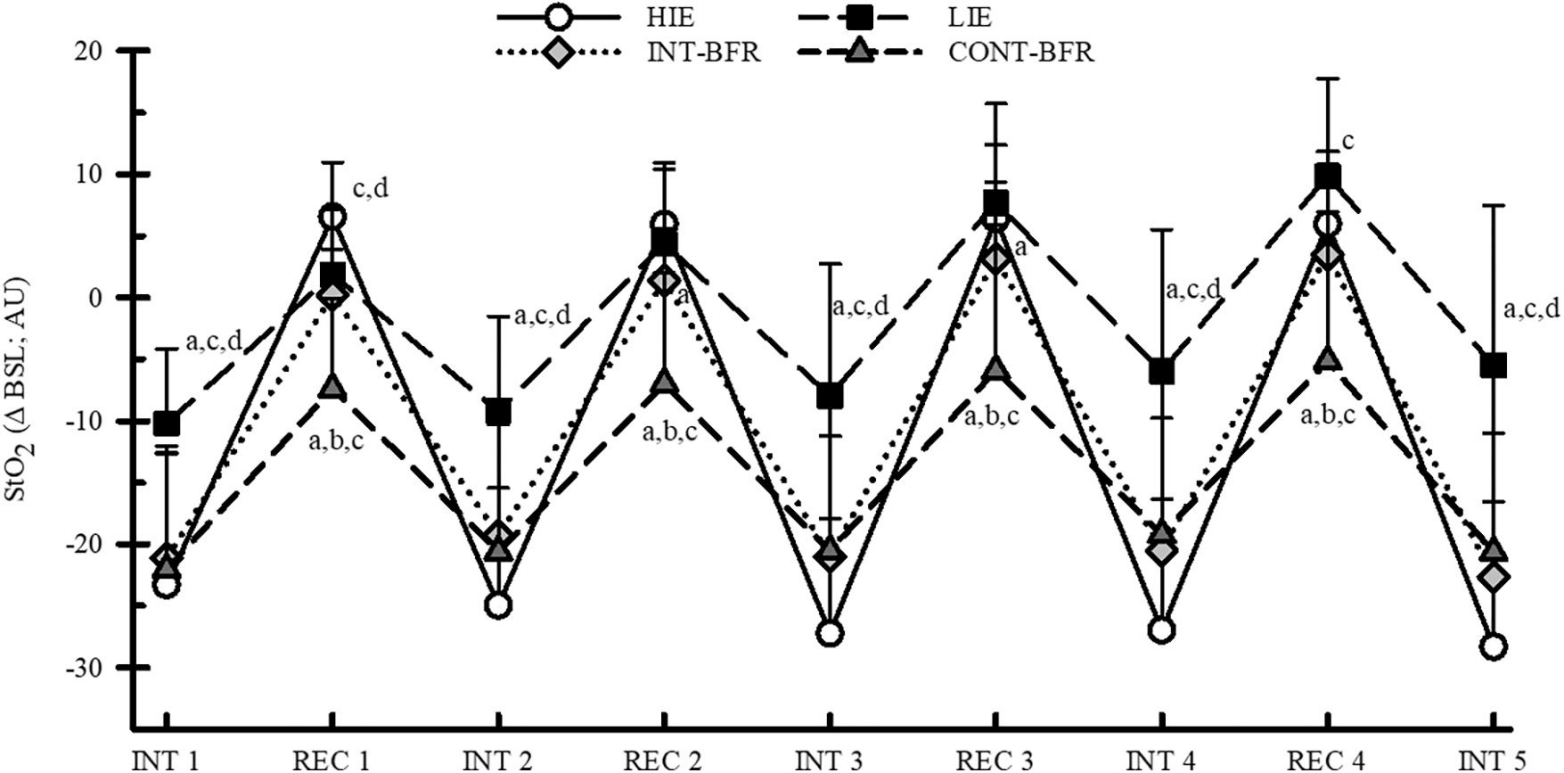
Tissue oxygen saturation (StO_2_) responses to the high-intensity (HIE) and low-intensity exercise without BFR (LIE) and low-intensity exercise with continuous (CONT-BFR) and intermittent BFR (INT-BFR) during work (INT) and recovery (REC) intervals. a = significantly different from HIE; b = significantly different from LIE c = significantly different from INTBFR; d = significantly different from CON-TBFR StO_2_ data are reported as the change from warm-up (ΔBSL) in arbitrary units (AU) and expressed as mean ± sd. Significance was established if *p* ≤ 0.05.

The StO_2_ responses during INTs and RECs for each condition are shown in Figure 1. There was a significant condition-by-time interaction for StO_2_ (*p* < 0.001; *η*_p_^2^ = 0.59). Moreover, StO₂ was significantly different in the LIE condition compared to all others (*p* ≤ 0.024 for all comparisons; d = 1.25–3.25). There were no differences between INT-BFR, CONT-BFR and HIE during INTs. During all RECs, StO_2_ during CONT-BFR was significantly different compared to all other conditions (*p* ≤ 0.036 for all comparisons, 1.24 ≤ d ≤ 2.60 for all comparisons). Furthermore, StO₂ differed between INT-BFR and HIE during REC 1 (*p* = 0.005, d = 1.02), REC 2 (*p* = 0.018, d = 0.91), and REC 3 (*p* = 0.016, d = 0.50). The difference during REC 4 did not reach significance (*p* = 0.08). The only other difference observed was between LIE and INT-BFR during REC 4 (*p* = 0.044, d = 1.07).

**Figure 2:**
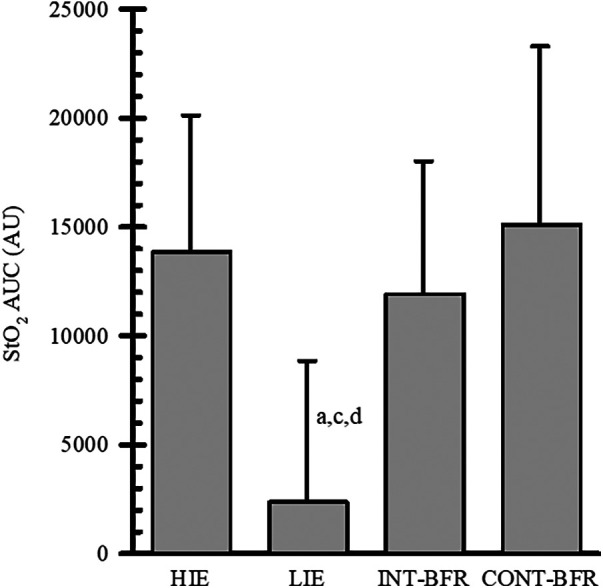
Tissue oxygen saturation (StO_2_) area under the curve (AUC) during the high-intensity (HIE) and low-intensity exercise without BFR (LIE) and low-intensity exercise with continuous (CONT-BFR) and intermittent BFR (INT-BFR) exercise protocols. a = significantly different from HIE; c = significantly different from INT-BFR; d = significantly different from CONTBFR. Data are expressed as mean ± sd. Significance was established if *p* ≤ 0.05.

The NIRS AUC for each condition is shown in Figure 2. The one-way ANOVA showed a significant main effect (*p* < 0.001; *η*_p_^2^ = 0.60). NIRS AUC was similar between INT-BFR, CONT-BFR, and HIE. However, all three conditions were significantly different from LIE (vs. HIE *p* = 0.008, d = 1.80; vs. INT-BFR *p* = 0.015, d = 1.52; vs. CONT-BFR *p* = 0.001, d = 2.03).

### 3.3 Cardiorespiratory responses

**Figure 3:**
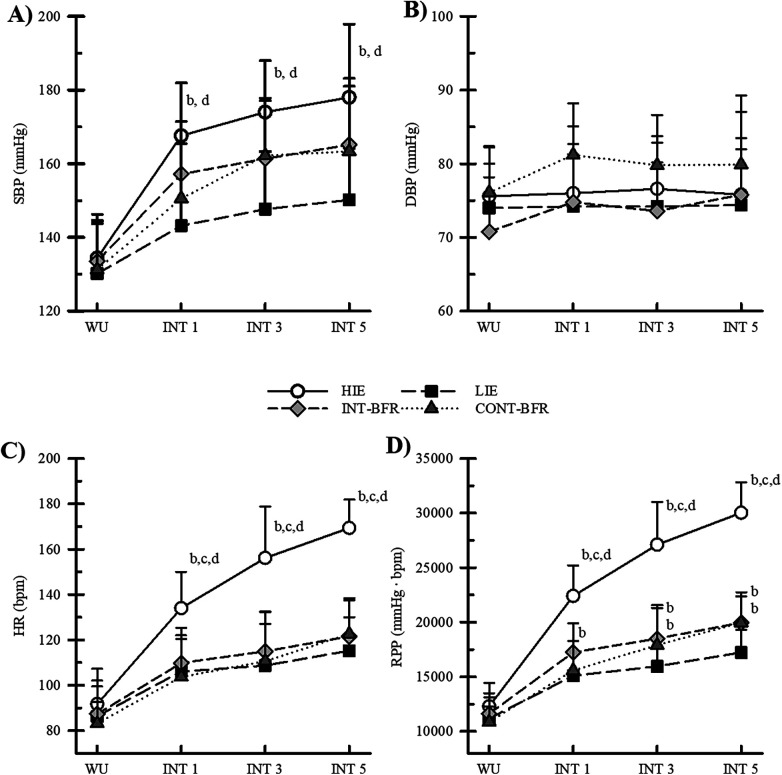
**(A)** Systolic blood pressure (SBP), **(B)** diastolic blood pressure (DBP), **(C)** heart rate (HR) and **(D)** rate pressure product (RPP) responses to high-intensity (HIE) and low-intensity exercise without BFR (LIE) and low-intensity exercise with continuous (CONT-BFR) and intermittent BFR (INT-BFR). a = significantly different from HIE; b = significantly different from LIE; c = significantly different from INT-BFR; d = significantly different from CONT-BFR. Data are expressed as mean ± sd. Significance was established if *p* ≤ 0.05. WU, warm-up; INT, work interval.

The VO_2_, CO, and SV responses to each condition are presented in Table 1. The HR responses to HIE, LIE, INT-BFR, and CONT-BFR are shown in Figure 3. There was a significant condition by time interaction for VO_2_ (*p* < 0.001; *η*_p_^2^ = 0.79), CO (*p* < 0.001; *η*_p_^2^ = 0.41), SV (*p* = 0.020, *η*_p_^2^ = 0.14), and HR (*p* < 0.001; *η*_p_^2^ = 0.60). There were no significant differences between conditions during WU for each variable (VO_2_ ICC = 0.514; CO ICC = 0.760; SV ICC = 0.912; SV ICC = 0.938); however, there were differences observed between conditions during INTs. Specifically, VO_2_ and HR during HIE were significantly greater than LIE, INT-BFR, and CONT-BFR during each interval (*p* < 0.01 for all comparisons, 1.69 ≤ d ≤ 4.99 for all comparisons). The only additional difference in VO₂ was observed during INT 3 between CONT-BFR and INT-BFR (*p* = 0.029, d = 0.61). During all INTs, CO was greater during HIE compared to INT-BFR and CONT-BFR (*p* ≤ 0.02 for all comparisons, 1.92 ≤ d ≤ 2.75 for all comparisons). Moreover, CO was greater during HIE compared to LIE during INT 3 (*p* = 0.008, d = 2.89), and INT 5 (*p* = 0.012, d = 2.57); the difference in CO during INT 1 did not reach significance (*p* = 0.08). Additionally, SV was similar among conditions during all INTs.

**Table 1:** Oxygen uptake, cardiac output and stroke volume responses.

Condition	VO_2_ (L·min−1)				CO (L·min−1)				SV (mL)			
	WU	INT 1	INT 3	INT 5	WU	INT 1	INT 3	INT 5	WU	INT 1	INT 3	INT 5
HIE	0.84 ± 0.05	1.90 ± 0.31	2.20 ± 0.25	2.25 ± 0.26	8.8 ± 1.7	15.4 ± 2.8	18.3 ± 4.1	19.8 ± 4.2	96.3 ± 18.5	112.6 ± 26.6	113.9 ± 31.7	117.3 ± 29.9
LIE	0.79 ± 0.05	1.31^a^ ± 0.19	1.32^a^ ± 0.13	1.34^a^ ± 0.17	8.4 ± 1.1	11.9 ± 2.1	12.8^a^ ± 2.0	13.4^a^ ± 1.5	97.6 ± 17.3	111.1 ± 20.7	115.6 ± 20.3	115.4 ± 23.6
INT-BFR	0.82 ± 0.11	1.28^a^ ± 0.26	1.29^a^ ± 0.17	1.33^a^ ± 0.15	8.3 ± 0.9	11.5^a^ ± 1.5	12.6^a^ ± 1.5	13.0^a^ ± 1.5	96.3 ± 19.3	102.7 ± 20.2	106.6 ± 21.6	106.5 ± 24.3
CONT-BFR	0.80 ± 0.07	1.31^a^ ± 0.17	1.40^a^^,^^b^ ± 0.16	1.40^a^ ± 0.20	7.8 ± 0.8	10.8^a^ ± 1.4	11.8^a^ ± 1.9	13.0^a^ ± 1.8	94.1 ± 17.1	102.2 ± 17.9	104.3 ± 22.8	104.8 ± 19.8

Data are expressed as mean ± sd. Significance was established if *p* ≤ 0.05. HIE, high-intensity exercise without BFR; LIE, low-intensity exercise without BFR; CONT-BFR, low-intensity exercise with continuous BFR; INT-BFR, low-intensity exercise with continuous BFR; WU, warm-up; INT, work interval; VO2, oxygen consumption; CO, cardiac output; SV, stroke volume.

a Significantly different from HIE.

b Significantly different from INT-BFR.

### 3.4 Blood pressure responses

The SBP, DBP, and RPP responses are shown in Figure 3. There was a significant condition-by-time interaction for SBP (*p* < 0.001; *η*_p_^2^ = 0.45) and RPP (*p* < 0.001; *η*_p_^2^ = 0.81). There were no significant effects of DBP (interaction effects *p* = 0.077; main effect of condition *p* = 0.055; main effect of time *p* = 0.387) observed. No differences in DBP (ICC = 0.856), SBP (ICC = 0.873), and RPP (ICC = 0.879) were observed between conditions during the WU. Additionally, SBP was similar among LIE, INT-BFR, and CONT-BFR during all INTs. However, SBP was greater in HIE compared to LIE and CONT-BFR during all INTs (for all comparisons *p* ≤ 0.032, 0.71 ≤ d ≤ 1.87). The differences in SBP between HIE and INT-BFR were not statistically significant.

There were no differences in RPP between INT-BFR and CONT-BFR. However, RPP was greater in HIE during all INTs compared to all other conditions (for all comparisons; *p* < 0.001, 2.32 ≤ d ≤ 5.63). In all work intervals, RPP during INT-BFR was greater than during LIE (for all comparisons; *p* ≤ 0.023, 1.18 ≤ d ≤ 1.39). There were also differences in RPP between LIE and CONT-BFR during INT 3 (*p* = 0.050, d = 0.95) and INT 5 (*p* = 0.005, d = 1.31); the difference in RPP during INT 1 was not significant.

## 4 Discussion

The primary purpose of this study was to determine the muscular hypoxic and cardiovascular responses to low-intensity aerobic exercise with continuous and intermittent BFR. This study found that both BFR protocols produced similar hypoxic stress during work intervals; however, CONT-BFR resulted in greater hypoxic stress than INT-BFR during recovery intervals. Despite differences during the recovery intervals, the overall hypoxic stress (i.e., StO_2_ AUC) was not different between the BFR conditions. Secondly, the restriction protocol (INT-BFR or CONT-BFR) did not alter myocardial work. These results suggest that the restriction protocol used during BFR exercise (i.e., CONT-BFR vs. INT-BFR) does not significantly alter muscle hypoxic or cardiovascular responses during work intervals of low-intensity aerobic exercise with BFR.

This study showed that StO_2_ responses during the work intervals were similar between INT-BFR, CONT-BFR, and HIE, and all three conditions produced a greater response compared to LIE. These findings suggest that the addition of BFR provided greater hypoxic stress to the exercising muscles compared to free-flow conditions at the same intensity and a similar hypoxic stress as the high-intensity condition. It is well established that low-intensity exercise with BFR increases local hypoxia compared to free-flow conditions of the same intensity and some studies have shown that low-intensity exercise with BFR can create a hypoxic stimulus comparable to high-intensity conditions (11, 27). Furthermore, the present study suggests that during low-intensity aerobic interval exercise with BFR, the restriction protocol (i.e., INT-BFR or CONT-BFR) does not affect the hypoxic stimulus during work intervals. Previous studies examining metabolic stress during intermittent and continuous BFR during resistance exercise support these findings (14, 15). In contrast, Corvino et al. (13) found that tissue oxygenation was lower during INT-BFR compared to CONT-BFR, suggesting greater hypoxia during INT-BFR. However, the Corvino study used a higher restriction pressure during the INT-BFR condition compared to CONT-BFR. Taken together with the present study, which used similar restriction pressures across protocols, these findings suggest that changes in StO_2_ during exercise with BFR can be attributed to the restriction pressure, not the restriction protocol or the duration of the restriction.

During active recovery intervals, StO_2_ responses during CONT-BFR were greater than INT-BFR, HIE, and LIE. Moreover, CONT-BFR was the only condition that StO_2_ was lower than warm-up during the recovery intervals, suggesting continuous hypoxia during the exercise protocol. Whereas in the other conditions, StO_2_ increased from work intervals to near or above the baseline during free-flow recovery intervals, suggesting reperfusion of blood to the exercising muscles. This result would be expected because restriction was maintained during recovery intervals during CONT-BFR, while recovery intervals in the other three protocols were under free-flowing conditions (11, 12, 28). Taken together, it appears that the restriction pressure could be the primary mediator of the StO_2_ response during aerobic interval exercise with BFR, and tissue hypoxia/blood reperfusion during recovery intervals does not affect the hypoxic stimulus during subsequent work intervals.

The hypoxic stimulus is thought to be important for the chronic adaptations in aerobic fitness and muscular strength and hypertrophy observed during BFR exercise training (2, 29). Chronic exposure to these stimuli has been shown to lead to positive changes in muscle oxidative function, primarily due to an increase in mitochondrial enzyme function and capillary density (30, 31). To understand the overall hypoxic stress of the conditions, the present study also examined the StO_2_ AUC. Although, the hypoxic stress during CONT-BFR was similar during work intervals and greater during recovery compared to INT-BFR and HIE, the overall hypoxic stress was not different between the three conditions. This appears to be the first study to show that the overall hypoxic stimulus during low-intensity aerobic exercise with BFR is similar to HIE. In support of this finding, Lauver et al. (11) found the average StO_2_ over the entire bout of exercise was similar between HIE and low-intensity aerobic exercise with BFR. It is currently unclear why INT-BFR and HIE produced a similar overall hypoxic stimulus compared to CONT-BFR. However, it is possible that the restriction did not create a large enough mismatch between oxygen delivery and oxygen demand during recovery intervals to create a large hypoxic stress. Regardless, acute hypoxic stress drives adaptations for improved aerobic fitness (2, 6) and was not different between INT-BFR and CONT-BFR. The acute responses suggest either restriction protocol may provide comparable chronic adaptations, but further research is needed to understand the effect of restriction protocol on chronic adaptations.

Furthermore, RPP was similar during the INT-BFR and CONT-BFR conditions in the present study. BFR protocols resulted in greater RPP compared to LIE, but lower than during HIE. Similar to these findings, studies in upright cycling and resistance exercise have consistently shown that the addition of BFR increases RPP compared to free-flowing conditions (5, 10, 14, 32). The increase in RPP with the addition of BFR is thought to be due to increases in contractility to compensate for the increased resistance to blood flow (8, 10). Supporting this assertion, the increase in myocardial work observed in this study during the BFR conditions compared to free-flowing conditions during low-intensity exercise was driven by the increase in SBP. When comparing myocardial work during low-intensity exercise with BFR and high-intensity exercise, the findings are more equivocal (10, 33). Some studies (32, 34, 35) have shown a greater increase in cardiac work during high-intensity exercise compared to low-intensity exercise with BFR, while, other studies have found similar increases in cardiac work (14, 33, 36). The differences in findings could be due to the large variations in methodology in the BFR literature, including differences in exercise intensity, exercise volume, and restriction pressures.

It was originally hypothesized that CONT-BFR would produce greater cardiac work compared to INT-BFR because continuous restriction increases metabolite accumulation and venous pooling resulting in increased SBP and/or an increase in HR to compensate for a lower SV (8, 37). However, it does not appear that restriction during the recovery intervals affected SBP, HR, and SV, as there were no differences between the BFR conditions. It is unclear why the cardiovascular responses were similar between CONT-BFR and INT-BFR. It is possible that the duration of free-flow conditions during INT-BFR was too short, or that restriction duration during work intervals allowed SBP and HR in INT-BFR to equal CONT-BFR when each were measured, but this remains unclear. Regardless, this appears to be the first study to show that continuous and intermittent BFR of the same restriction pressure produces similar increases in myocardial work during aerobic exercise.

The VO_2_ and CO were greatest in the HIE condition, but there were no other differences between the conditions. These results agree with other studies that have examined BFR during cycling (11, 13, 34, 35). Specifically, Lauver et al. (11) showed VO_2_ was not different during free flow and BFR cycling at similar work rates, but differences in VO_2_ were observed with different work rates. However, not all studies have found similar VO_2_ with the addition of BFR (5, 38). The reason for the variation in results is unclear but could be due to differences in protocols. This study shows that the restriction protocol (e.g., CONT-BFR or INT-BFR) during aerobic exercise with BFR does not affect the oxidative energy demand, which appears to be primarily regulated by work rate, independent of BFR.

This study had limitations, as it only examined healthy, young male individuals, making it difficult to generalize the findings to women and older or clinical populations. Although cardiovascular responses to BFR vs. free-flow conditions may differ across populations, there is no evidence suggesting that the restriction protocol alters these acute responses. Additionally, this study manually measured arterial blood pressure to assess cardiovascular stress because RPP is a clinical measurement that is an indirect measurement of cardiovascular work. However, the blood pressure response during RECs could not be ascertained due to the nature of the manual blood pressure measurement. Future research using continuous blood pressure monitoring is needed to better understand cardiovascular responses during INT-BFR and CONT-BFR aerobic exercise protocols.

## 5 Conclusion

The primary finding from this study was that when adding BFR to low-intensity aerobic exercise the restriction protocol (CONT-BFR vs. INT-BFR) did not affect muscle hypoxia or cardiovascular work. Specifically, the addition of BFR increased the overall muscular hypoxic stress compared to free-flow conditions, but the hypoxic stress was similar between BFR conditions and the HIE condition. Similarly, there were no differences in cardiac work between restriction protocols, but BFR conditions produced less cardiac work compared to the HIE condition. Taken together, these results suggest that the addition of BFR, regardless of restriction protocol, produces a similar local hypoxic stress to high-intensity aerobic exercise, without increasing cardiovascular stress. This suggests that the restriction protocol does not affect muscular or cardiovascular stress and low-intensity aerobic interval exercise with BFR, regardless of restriction protocol, could be a viable alternative to high-intensity interval exercise.

## Data Availability

The raw data supporting the conclusions of this article will be made available by the authors, without undue reservation.
